# The effect of ileal resection length on postoperative complications and prognosis in right colon cancer

**DOI:** 10.1007/s00423-024-03395-9

**Published:** 2024-07-05

**Authors:** Murat Yıldırım, Asım Kocabay, Bulent Koca, Ali Ihsan Saglam, Namık Ozkan

**Affiliations:** Department of General Surgery, Faculty of Medicine, Gaziosmanpasa University, Sevki Erek Yerleskesi, Tokat, 60030 Turkey

**Keywords:** Right hemicolectomy, Terminal ileum, Colon cancer, Overall survival, Disease-free survival

## Abstract

**Background:**

There is a lack of literature on the length of the terminal ileum to be resected in right hemicolectomy for colon cancer. Therefore, we aimed to determine the mean ileal loop length and the effect of this variation on postoperative complications and long-term oncological outcomes in patients who underwent right hemicolectomy.

**Methods:**

Right hemicolectomy surgeries performed for colon cancer in a tertiary care hospital between January 2011 and December 2018 were retrospectively analyzed from a prospective database. Two patient groups were established based on the mean length of the resected ileum above and below 7 cm. The two groups were compared for clinicopathological data, postoperative complications, mortality, long-term overall survival (OS) and disease-free survival (DFS). The factors contributing to OS and DFS were analyzed.

**Results:**

The study included 217 patients. Body mass index (BMI) values were significantly higher in the ileum resection length > 7 cm group (*p* = 0.009). Pathological N stage, tumor diameter, and number of metastatic lymph nodes were significantly higher in the ileum resection length > 7 cm group (*p* = 0.001, *p* = 0.001, and *p* = 0.026, respectively). There was no significant difference for postoperative complication and mortality rates between the two groups. The mean follow-up period was 61.2 months (2-120) in all patients. The total number of deaths was 29 (11.7%) while the 60-month OS was 83.5% and 50-month DFS was 81.8%. There was no significant difference between the groups in terms of OS and DFS rates (*p* > 0.05).

**Conclusions:**

Excessive resection of the distal ileum in right hemicolectomy does not provide any benefit in terms of prognosis and complications.The ileum resection length and values close to it in our study appear to be sufficient.

## Introduction

The standard surgical procedure in right colon cancer involves adequate lymph node dissection with resection of part of the distal ileum and the right colon and is called right hemicolectomy [1]. With adequate lymph node dissection, it is possible to remove the lymph nodes around the ileocolic, right colic and middle colic arteries and ligation of these vessels because lymph node metastases occur along these main vessels. Similarly, lymph node metastases around the distal ileum and mesentery of terminal ileum are also possible.

In addition, nutritional disorders that occur with right colon resection may lead to further complications and malnutrition with excessive terminal ileum resection. Bile salts and vitamin B12 are absorbed in the terminal ileum. Therefore, vitamin and mineral malabsorption is inevitable after terminal ileal resection with severe diarrhea [2, 3]. The prevalence rate of bile acid malabsorption following terminal ileal resection and right hemicolectomy was reported to be around 90%,and this can occur after resection of a small part of the terminal ileum as small as 10 cm [4]. Although surgeons want to remove the terminal ileum more extensivelybased on tumor localization in terms of oncological safety concerns, there is no consensus in the literature about how long this length should be.

The aim of the present study was to investigate the effect of terminal ileum resection length on postoperative complications and survival in patients who underwent right hemicolectomy for colon cancer.

## Materials and methods

Right hemicolectomy surgeries performed for colon cancer in the oncological surgery clinic of Tokat Gaziosmanpaşa University Education and Research Center between January 2011 and December 2018 were retrospectively analyzed from the prospective database.

Patients under 18 years of age, patients with distant organ and/or peritoneal metastasis (stage 4), patients considered unresectable at the time of surgery, patients who underwent emergency surgery, patients with acute liver and kidney failure and cirrhosis, and patients with missing records were excluded from the study. Since our hospital is located in a rural region of our country, unfortunately, we did not have a medical oncologist in some periods. During these periods, adjuvant therapy could be performed in other towns for logistical reasons. These patients were also excluded from the study.

The diagnosis of colon cancer was preoperatively confirmed histopathologically confirmed adenocarcinoma. The patients were staged with multi-slice abdominal and thoracic CT preoperatively. PET/CT was used in necessary cases. The decision to perform operation was taken by the multidisciplinary tumor council, which is held weekly in our clinic. The surgeries were performed by senior colorectal surgeons using a laparoscopic or open approach. Mechanical bowel cleansing was performed in all patients. The patients were administered a pre-prophylactic antibiotic (1 g cefazolin intravenously) 30 min before the operation and as an additional dose for patients with a duration exceeding three hours. All patients were admitted to the postoperative intensive care unit. The patients in good general condition were transferred to the general surgery service the next day. Postoperative antibiotics (2nd or 3rd generation cephalosporin and metronidazole, intravenous) and low molecular weight heparin were administered to all patients. The surgical procedure included at least D2 lymphadenectomy and the standard right hemicolectomy procedure.

Demographic data (age, gender), American Society of Anesthesiologists (ASA) status, preoperative body mass index (BMI), operative details (laparoscopic/open technique, intraoperative complication, blood transfusion), pathology results, ileal resection length, recurrence status, survival, oncological follow-up data, postoperative complications were prospectively collected in a database, anonymized and then analyzed.

Postoperative complications were defined as surgical and non-surgical complications from the postoperative period until discharge. The Clavien-Dindo classification system was used to classify all postoperative complications [5]. The patients were divided into two groups according to the presence of postoperative major (stage III and more according to the Clavien-Dindo classification) and minor complications (stage I/II or no complications). Mortality was defined as death within 30 days from the date of surgery.

Recurrence was defined as the appearance of new lesions in the anastomosis and/or surrounding colon wall and/or in the lymphatic drainage zone of the previously resected tumor in the postoperative period, confirmed by clinical, scanning tomography and positron emission tomography-CT (PET-CT) or pathological examination. Recurrence of the disease in the peritoneum or other organs was considered as distant metastasis.

The mean ileal resection length obtained from the pathological reports of the patients was calculated as 7 cm (1–18) using arithmetic analysis. We also performed a statistical cut-off analysis to prevent it from being considered as an arbitrary length. We found the mean cut-off value for terminal ileum to be 6.83 cm and used 7 cm because it was close to our mean value. The patients were divided into two groups according to the length of the ileum material: group 1 (≤ 7 cm) and group 2 (> 7 cm).

### Statistical analysis

Statistical analysis of the data obtained in this study was performed using SPSS software (Version 22, SPSS Inc., Chicago, IL, USA). Data are expressed as mean ± standard deviation. Student’s t-test was used to analyze quantitative variables, and the χ2 test was used for qualitative ones. Overall survival (OS) and disease-free survival (DFS) survival curves were prepared according to the Kaplan–Meier method and statistical differences were determinedusing the log-rank test. Univariate and multivariate Cox-proportional hazard models were used to identify risk factors for OS and DFS. *P* value < 0.05 was considered to indicate statistical significance.

## Results

There were 248 patients who underwent right hemicolectomy for colon cancer. Eighteen patients with stage 4 disease, 11 patients with missing data and who did not accept adjuvant therapy, 2 patients with concomitant Crohn’s disease, and 1 patient who died during surgery due to cardiac reasons were excluded. As a result, we had data for a total of 216 patients who were operated for stage 1–3 right colon tumors. There were 97 patients with ≤ 7 cm ileum resection length (group 1) and 119 patients with > 7 cm ileum resection length (group 2). Demographic and clinicopathological data of the two groups are shown in Tables 1 and 2.

**Table 1:** Demographic data of the study groups

	Group1(*n*:97)	Group2(*n*:119)	*p*-value
Age (years)(mean ± SD)	66.59 ± 14.5	65.87 ± 11.9	0.69
Gender (n,%)			0.723
Female Male	49(50.5) 48(49.4)	63(52.9) 56(47)	
BMI(mean ± SD)	26.83 ± 4.9	28.94 ± 6.5	**0.009**
Location of cancer, *n* (%)			0.14
Caecum Ascending colon Transverse colon	44(45.3) 33(34) 20(20)	73(61.3) 36(30.2) 10(8.4)	
ASA(n,%)			0.16
1 2 3 4	5(5.1) 24(24.7) 57(58.7) 11(11.3)	1(0.8) 24(20.1) 63(52.9) 31(26.0)	
Surgical technique (n,%)			**0.008**
Laparoscopic Open Converted to open	50(51.5) 40(41.2) 7(7.2)	40(33.6) 71(59.6) 8(6.7)	
Duration of hospitalization (day) (mean ± SD)	11.07 ± 8.79	11.74 ± 12.66	0.657
ICU length of stay(day) (mean ± SD)	2.29 ± 2.97	3.14 ± 6.48	0.238

BMI: Body Mass Index, ICU: Intensive Care Unit

**Table 2:** Pathological data, complications and mortality rates of the study groups

	Group1(*n*:97)	Group2(*n*:119)	*P* value
T Stage (n,%)			0.229
1 2 3 4	11(11.3) 30(30.9) 49(50.5) 7(7.2)	8(6.7) 33(27.7) 74(62.1) 4(3.3)	
N Stage(n,%)			**0.001**
0 1 2	57(58.7) 29(29.8) 11(11.3)	63(52.9) 19(15.9) 37(31.1)	
Perineural invasion (*n,%)*)			0.377
Yes No	34(35) 63(64.9)	35(29.4) 84(70.5)	
Lymphatic invasion (*n,%*)			0.724
Yes No	24(24.7) 73(75)	27(22.6) 92(77.3)	
Vascular invasion (*n,%*)			0.546
Yes No	22(22.6) 75(77.3)	23(19.3) 96(80.6)	
Tumor diameter	4.46 ± 2.02	6.32 ± 3.7	**0.001**
Number of lymph nodes	20.54 ± 8.26	21.51 ± 9.1	0.42
Number of metastaticlymph nodes	1.98 ± 2.57	3.02 ± 3.89	**0.026**
Postoperative Complication			0.395
No/Minor(*n,%)*) Major (*n,%)*)	89(91.7) 8(8.2)	105(88.2) 14(11.7)	
Mortality	4(4.1)	5(4.2)	-

The mean age in group 1 was 66.59 ± 14.5 years, 49 patients were female and 48 were male in the group, while the mean age in group 2 was 65.87 ± 11.9 years, and there were 63 females and 56 males. There was no significant difference between the groups for gender distribution and mean age (*p* > 0.05). BMI values were significantly higher in group 2 (*p* = 0.009). In terms of the surgical procedure type, number of operations with laparoscopic technique was higher in group 1 while open technique was used more in group 2, and there was a significant difference between the two groups (*p* = 0.008). There was no significant difference between cancer location, ASA scores, length of hospital and intensive care unit stay between the two groups.

Pathological N stage, tumor diameter, and number of metastatic lymph nodes were significantly higher in the ileum length > 7 cm group (*P* = 0.001, *P* = 0.001, and *P* = 0.026, respectively). There was no significant difference in terms of T stage, perineural invasion, vascular invasion, lymphatic invasion and lymph node count (*p* > 0.05).

There was no significant difference between the two groups in terms of major and minor/no complication rates. A total of 22 patients (10.1%) had major complications. Major complications were anastomotic leakage in 6 (2.7%), ileus in 11 (5%), intra-abdominal abscess in 4 (1.8%), intra-abdominal hemorrhage in 2 (0.9%), and evisceration at the wound site in 3 (1.3%) patients. The overall mortality rate was 4.1% (*n* = 4) in group 1 and 4.2% (*n* = 5) in group 2, and there was no significant difference between the two groups (*P* = 0.3).

The mean follow-up period in all patients was 61.2 months (2-120). The total number of deaths was 29 (11.7%), 60-month overall survival was 83.5%, and 50-month disease-free survival was 81.8%.

**Fig. 1:**
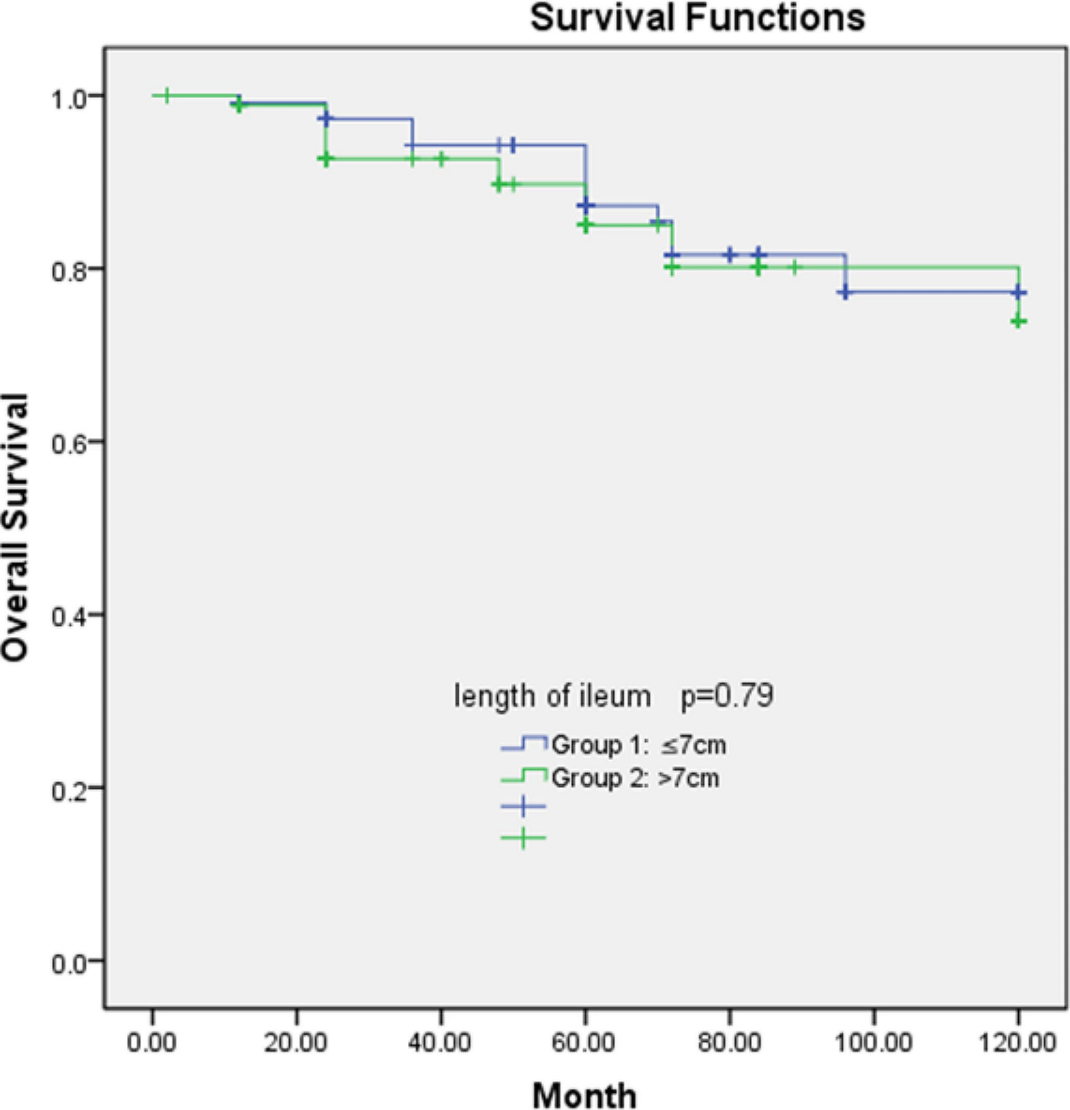
Association of overall survival rate and ileum resection length

**Fig. 2:**
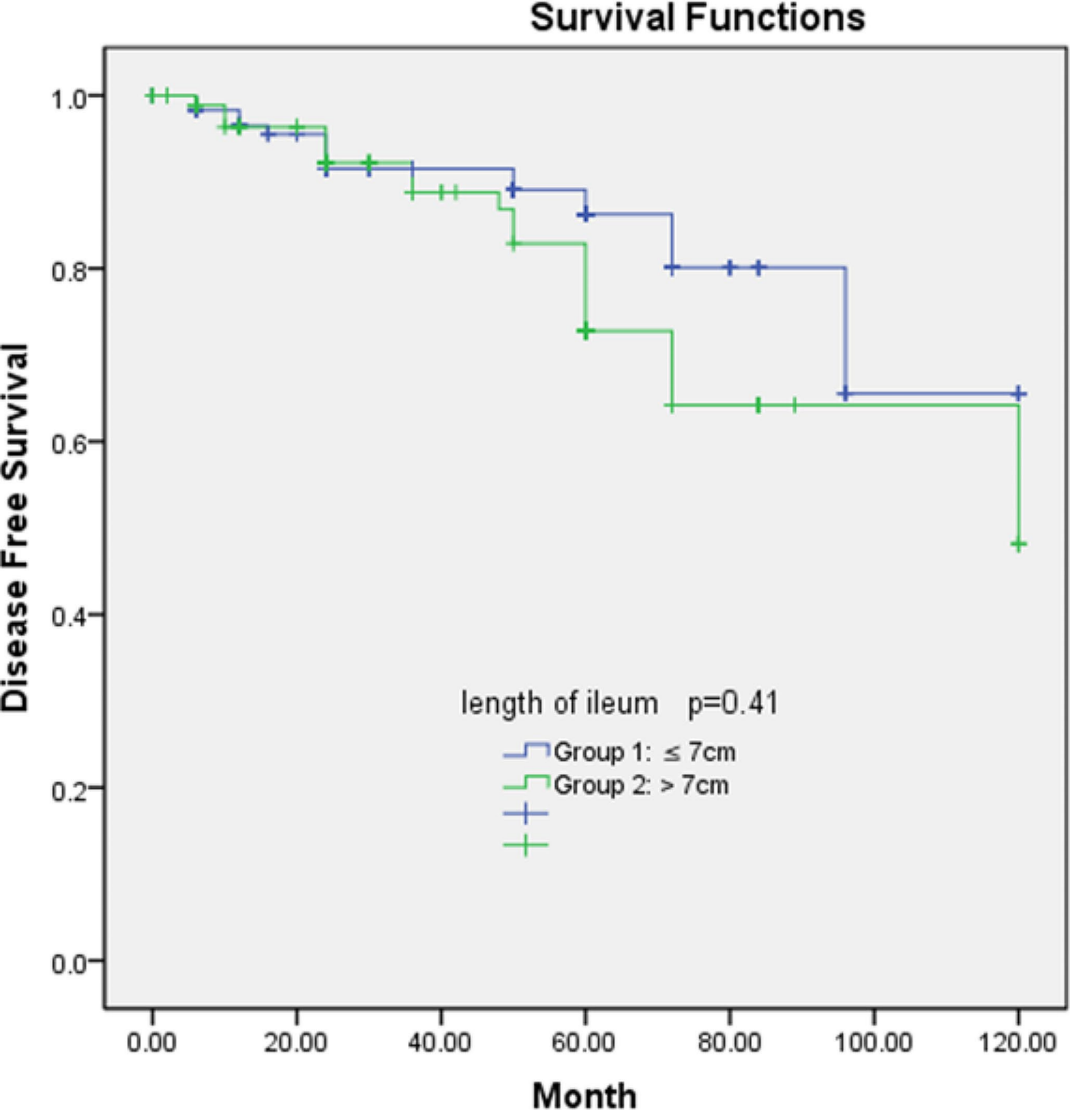
Association of disease-free survival and ileum resection length

Overall survival and disease-free survival rates of the two groups are shown in Figs. 1 and 2. There was no significant difference between the groups in terms of overall survival (*P* = 0.79) and disease-free survival (*P* = 0.41). The 5-year overall survival rates of the groups 1 and 2 were 84.1% and 75.9%, respectively while the 5-year disease-free survival rates were 83.8% and 78.3%, respectively.

T stage (*P* = 0.01), N stage (*P* = 0.009) and metastatic lymph node (*P* = 0.03) status were significantly correlated in univariate analysis for OS. Similarly, T stage (95% CI = 0.485–0.953, *P* = 0.01), N stage (95% CI = 0.154–0.897, *P* = 0.01) and metastatic lymph node (95% CI = 0.398-1.1, *P* = 0.03) were also correlated in multivariate analysis (Table 3).

**Table 3:** Univariate and multivariate analysis of clinic-pathological data and overall survival

	Univariate analysis		Multivariate analysis	
	(CI 95%)	*P* values	(CI 95%)	*P* values
Age		0.375		
Gender		0.733		
BMI		0.575		
ASA		0.71		
Tumor location		0.09		
Type of the operation		0.91		
T stage	(101.5-110.8)	***0.012***	**(0.485–0.953)**	**0.01**
N stage	(101.8-110.6)	***0.009***	**(0.154–0.897)**	**0.01**
LI		0.253		
PNI		0.602		
VI		0.362		
Metastatic lymph node	(101.8-110.6)	**0.031**	**(0.398-1.1)**	**0.03**
Number of lymph nodes		0.253		

LI: Lymphatic invasion, PNI: Perineural invasion, VI: Vascular invasion, BMI: Body Mass Index

For DFS, operation type (*P* = 0.01), N stage (*P* = 0.04), T stage (*P* = 0.02) and metastatic lymph node status (*P* = 0.01) were significantly correlated in univariate analysis. However, in multivariate analysis, only metastatic lymph node status was significantly associated (95% CI = 0.185–0.804, *P* = 0.01) (Table 4).

**Table 4:** Univariate and multivariate analysis of clinic-pathological data and DFS

	Univariate analysis (CI 95%)	*P* values	Multivariate analysis (CI 95%)	*P* values
Age		0.273		
Gender		0.232		
BMI		0.630		
ASA		0.14		
Tumor location		0.202		
Type of the operation	**(91.9–104)**	**0.018**	(096 − 3.8)	0.65
T stage	**(91.9-104.9)**	***0.04***	(0.81–1.78)	0.22
N stage	(**91.9-104.9**)	***0.02***	(0.596–3.72)	0.39
LI		0.06		
PNI		0.501		
VI		0.06		
Metastatic lymph node	(91.6-104.9)	**0.01**	**(0.185–0.804)**	**0.01**
Number of lymph nodes		0.253		

LI: Lymphatic invasion, PNI: Perineural invasion, VI: Vascular invasion, BMI: Body Mass Index

## Discussion

In our study, the terminal ileum lengths were different in patients who underwent right hemicolectomy for colon cancer. We did not find a significant difference for overall survival and disease-free survival between the two ileum resection groups of above and below 7 cm long. Postoperative complication rates of the two groups were similar. Tumor diameter, T stage, and number of metastatic lymph nodes were significantly larger in the group with long ileal resection.

Intestinal dysfunctions that may occur after rectal surgery and their effects on the gastrointestinal system are well documented [6, 7]. However, the effect of right hemicolectomy on bowel function has been less addressed. Disruption of the ileocecal valve and resection of the terminal ileum of different lengths are the most important factors in the deterioration of gastrointestinal functions in right hemicolectomy. There is not enough information in international guidelines about the extent of ileal resection in right hemicolectomy. Several studies focused on inflammatory bowel diseases in general and Crohn’s disease in particular [8–10]. In these studies, gastrointestinal dysfunctions such as bile acid malabsorption, chronic diarrhea, and B12malabsorption after ileal resection were well explained and it was emphasized that they correlated with the length of the removed terminal ileum.

In addition, the length of the ileum to be removed in right hemicolectomies performed due to cancer diagnosis is not clear in the literature. It is generally recommended to resect the proximal and distal to the tumor with an intestinal margin of 5 to 10 cm [11]. Studies focused on the lymph nodes around the primary feeder artery rather than the length of the ileum. In recent years, the concept of complete mesocolic excision (CME), involving the complete removal of the visceral peritoneum and mesentery, which includes the lymph nodes of the tumor area that perform regional drainage, has been accepted [12, 13]. However, even this issue has not been clarified. Although most clinics target lymph nodes around the superior mesenteric vein, the extent of lymphadenectomy is not clarified in American and European guidelines [14]. Within the scope of our study, we did not distinguish between patients who underwent CME and those who did not, but in recent years, we have been paying attention to perform CME in colon surgery in our clinic.

However, metastatic lymph nodes can also be found in the mesentery of the terminal ileum which is fed by ileocolic vessels, as well as central lymph nodes. In their retrospective study on 275 patients with right colon cancer, Toyota et al. [15] detected metastases in the peri-ileal lymph nodes in eight patients with cecum cancer. Their average distance to ileocecal valve. In a recent study in which metastatic features of the terminal ileum and infrapyloric lymph nodes were analyzed in patients with right colon cancer, the patients were divided into three groups as ileocecal, ascending colon and hepatic flexure group, and distribution of lymph node metastases were examined. The total number of dissected terminal ileum lymph nodes was 253, of which 17 (6.7%) were metastatic. The researchers did not provide information about the length of the dissected terminal ileum. However, they performed extended resection of the terminal ileum in cecum cancer patients [16]. Baik et al. reported that 10 cm ileum resection would be sufficient in patients with cecum cancer [17].

In the present study, BMI was significantly higher in the group with a mean terminal ileum length of more than 7 cm. This was consistent withthe study by Ismail et al. [18] involving 50 patients with right hemicolectomy, in which the average ileum length was 5 cm. The researchers emphasized that the reason for this may be due to technical reasons in obese patients. We agree with this view. In obese and mesentery fatty patients, the ileum may be left long due to safety concerns regarding anastomosis.

In the present study, there was no significant difference for OS and DFS between the groups above and below 7 cm, which is the mean ileum variation. Studies on this in the literature are extremely limited. In their study, Baik et al. [17] reported the ileum length as 10 cm in locally advanced, stage 2–3 cecum cancers, and the 5-year survival did not differ significantly in both groups. The authors reported that removal of the ileum longer than 10 cm did not contribute to oncological outcomes in stage 2 and stage 3 cecum cancer.

Our study was comparable to the literature with 10.1% early postoperative major complication and 4.1% mortality rates [19, 20]. The most common postoperative major complications were postoperative ileus and anastomotic leakage. However, we did not find a significant association between the length of the removed ileum and postoperative complications and mortality.

Few studies reported long-term oncological outcomes after right hemicolectomy. However, a recent meta-analysisreported that the data from those studies were not statistically comparable and that the included studies were highly heterogeneous [21]. In this respect, with our 83.5% sixty-month overall survival and 81.8% fifty-month disease-free survival rates, our study contributes to the literature on the issue. However, in our study, we did not compare patients with CME and conventional right hemicolectomy. In recent years, the literature has focused on CME and non-CME oncological outcomes [22–24].

The retrospective design of our study had the inherent limitations of the design, we could not investigate the effect of terminal ileum length on bowel functions. This may be a different subject of study. Some patients may have been missed due to the coding error. In addition, the study utilized a single center data, which needs to be confirmed with multicentric prospective randomized controlled trials. However, there are very few studies on this subject. We think that the present study could contribute to the literature with this feature.

## Conclusion

Excessive resection of the distal ileum due to oncological safety concerns in right hemicolectomy does not provide any benefit in terms of prognosis and complications.We are of the opinion that the average ileum resection length and values close to it in the present study are sufficient. We believe that clinicians should keep in mind that gastrointestinal dysfunction, which is negatively affected by the disruption of the ileocecal valve, may increase further.

## Data Availability

No datasets were generated or analysed during the current study.
